# Recent trends of infant motor activity estimation using wearable sensors: A scoping review

**DOI:** 10.1371/journal.pdig.0001809

**Published:** 2026-11-06

**Authors:** Alexa DeVito, Ketaki Inamdar, Margaret E. O'Neil, Yidong Zhu, Mohammad Arif Ul Alam, Ainat Koren

**Affiliations:** 1 Department of Biomedical Engineering, University of Massachusetts Lowell, Lowell, Massachusetts, United States of America; 2 Division of Physical Therapy, Human Performance, School of Medicine, West Virginia University, Morgantown, West Virginia, United States of America; 3 Department of Physical Therapy and Kinesiology, Zuckerberg College of Health Sciences, University of Massachusetts, Lowell, Massachusetts, United States of America; 4 Miner School of Computer and Information Sciences, University of Massachusetts, Lowell, Massachusetts, United States of America; 5 Solomont School of Nursing, University of Massachusetts, Lowell, Massachusetts, United States of America; Independant, FRANCE

## Abstract

Wearable sensors may enable objective monitoring of infant motor activity, posture, and movement in laboratory, clinic, and home contexts. However, evidence for the validity, generalizability, and implementation readiness of wearable-derived infant motor measures remains heterogeneous. This scoping review synthesized technical features, validation evidence, and clinical applications of wearable sensors monitoring infant motor activity, posture, or movement during awake periods in the first year of life. PRISMA-ScR guidelines were followed, and the protocol was prospectively registered on Open Science Framework. Searches for peer-reviewed articles on wearable-based monitoring of infant activity published between 2019 and 2024 were identified through searches of PubMed, CINAHL, IEEE Xplore, and the ACM Digital Library. A total of 41 studies were selected and classified using Keadle's five-phase validation framework, distinguishing technical validation (Phases I–III) from applied implementation (Phase IV). Fifteen studies (36.6%) included typically developing infants, 22 (53.7%) included infants with or at-risk for developmental disabilities, and 4 (9.8%) were unspecified. Fifteen studies (36.6%) focused on technical validation; 26 (63.4%) implemented sensors in applied contexts. Triaxial accelerometers (n = 15) and IMUs (n = 24) were most common, with sampling frequencies from 1–150 Hz. Posture classification accuracy was 95.8%–98.2% in studies directly comparing posture and movement, whereas fine-grained movement classification was near 81%–84%. Reference-standard correspondence was generally strong for accumulated posture duration (r = 0.80–0.99) but more variable for movement and clinical measures (r = 0.61–0.98). Application studies quantified physical activity, movement coordination and complexity, and intervention responsiveness, with over half collecting home-based data. Wearable sensors have evolved from proof-of-concept systems to family-centered digital health tools supporting developmental monitoring and physical activity promotion. When paired with caregiver feedback and infant-centered design, these technologies may support families, complement clinic-based assessment, and extend developmental support access. Future research should prioritize standardized reporting, affordability, and open-source analytic pipelines to advance equitable implementation.

## Author summary

Infant movement provides important information about early health and development, but it is difficult to measure continuously during everyday life. Wearable sensors offer a way to monitor infant activity and posture outside the clinic, including in the home. In this review, we examined 41 studies published between 2019 and 2024 that used wearable sensors to measure movement during the first year of life. We found that accelerometers and other motion-sensing devices could often identify infant posture and gross activities with high accuracy. Researchers also used these technologies to study physical activity, movement coordination, developmental differences, and responses to intervention. More than half of the applied studies collected data in home settings, showing growing interest in real-world use. However, differences in devices, methods, reporting, affordability, and data analysis continue to limit comparison and widespread adoption. Wearable sensors may eventually help families and healthcare professionals monitor development over longer periods and complement clinic-based assessments, but more consistent, accessible, and infant-centered approaches are needed.

## Introduction

The first year of life is a critical period for motor development, during which infants develop postural control and movement skills that form the foundation for later physical function. Regular infant motor activity, including supervised awake play, supports this development [1]. Among these activities, tummy time, involving supervised play while infants are positioned in prone, strengthens extensor muscles (spine, shoulder, and hips) needed to achieve motor milestones like rolling and crawling [1,2]. Tummy time activity also reduces the risk of plagiocephaly a positional skull deformity (found in children who spend increased time in supine) and encourages the developmental motor sequence [2–4]. From a digital health perspective, wearable sensors offer accessible, remote approaches for monitoring infant movement, extending assessment and guidance beyond clinic-based encounters and supporting family-centered care.

Wearable sensor technologies, including accelerometers and intelligent multi-sensor suits, represent a significant advancement in digital health infrastructure by enabling objective, reliable monitoring of infant motor activity and posture during tummy time and other awake periods [5,6]. These devices can quantify time spent in different positions, as well as the duration and frequency of movement, to support assessment of motor development and identification of atypical movement patterns [2,7]. The integration of machine learning algorithms further supports high-fidelity analysis of movement data, enhancing early identification of motor issues and intervention opportunities [7]. Applications linked to these devices provide actionable feedback and alerts, empowering families to encourage safe, frequent motor experiences for infant growth and development [8]. Wearable sensors are not intended to replace clinical assessment but can complement clinical assessments by providing objective, longitudinal movement data to inform clinical decision-making and plans of care [5].

Evidence is growing in this field of study as indicated by the number of published sensor-based articles for infants [6]. However, the literature does not include many reviews that integrate both the state of the science on monitor development and application of objective motor measures for infants with and without disabilities. The aim of this scoping review is to summarize technical features and use of wearable sensors to monitor or quantify infant motor activity, posture, or movement during awake periods in the first year of life among infants with typical development and infants with developmental delays or disabilities. This review was conducted by a multidisciplinary team representing Nursing, Physical Therapy, Computer Science, and Biomedical Engineering. It summarizes the state of the science and provides an update on current developments, standardization and validation, to guide future developers and foster collaboration between clinical and technical research applications.

## Methods

### Review registration

The protocol was registered prospectively in Open Science Framework (https://osf.io/s384k) and this review was conducted in alignment with the protocol. The Preferred Reporting Items for Systematic Reviews and Meta-Analyses extension for Scoping Reviews (PRISMA-ScR) guidelines were followed. A completed PRISMA-ScR checklist is provided in S1 Table.

### Search strategy

A comprehensive search was conducted across several databases (PubMed, CINAHL, IEEE Xplore, and the ACM Digital Library) to identify relevant studies published between 2019 and 2024. These databases were selected to capture literature spanning both clinical and engineering disciplines related to research on wearable sensors to measure infant movement. The search strategy was developed in consultation with a research librarian. A detailed search strategy for PubMed is provided in S2 Table. The same strategy was applied to CINAHL and IEEE Xplore, with minor adaptations for the ACM Digital Library to account for database-specific indexing. Because this review did not evaluate an intervention, a modified PICOS framework was used to structure the search and screening process, focusing on Population, Indicators, Comparators (where applicable), Outcomes, and Study Design. The search combined Medical Subject Headings (MeSH) and relevant keywords. Core MeSH terms included “infant”, “exercise”, “patient positioning”, and “movement”, which were combined with keywords such as “tummy time”, “wearable sensors”, and “activity monitoring.” Within each concept group, terms were connected using the Boolean operator OR, and the major concept groups were linked using AND.

### Eligibility criteria

#### Inclusion criteria.

**1) Population:** Studies including infants aged 0–12 months, with or without developmental delay or disability, were eligible for inclusion. Studies enrolling broader age ranges were included if at least 50% of participants fell within the 0–12-month range or if data for this subgroup were reported separately. Studies from all geographical regions and settings were considered, including neonatal intensive care units, clinics, homes, and laboratory environments, **2) Indicators (Exposure):** Eligible studies examined wearable sensors used to monitor or quantify infant motor activity, posture, or movement during awake periods. Sensors could include accelerometers, inertial measurement units (IMUs), or other body-worn motion sensors. (including clothing or fabric with embedded sensors), **3) Comparators**: Comparators were not required for inclusion. However, when available, studies that compared sensor-based data with reference standards, observational assessments, or other measurement tools were included to evaluate validity or reliability of the sensors against the defined ‘gold standard’ measures, **4) Outcomes**: Outcomes included the quantification of movement or posture, assessment of developmental or motor indicators, adherence to positional recommendations such as tummy time, and evaluation of the accuracy, validity, or reliability of the wearable device in measuring these outcomes, **5) Study Design**: Eligible designs included peer-reviewed empirical studies and validation or feasibility studies presenting original data. Acceptable study designs were randomized controlled trials, cohort studies, case–control studies, cross-sectional studies, and case reports. We included studies published from 2019 to 2024 to capture contemporary evidence from the most recent five-year period, during which wearable-sensor hardware, signal-processing methods, and machine-learning applications for infant monitoring have advanced substantially. This time frame also aligned with publication of the Keadle framework, which we used to classify the developmental phase of body-worn-device studies (Phases I–IV) [9]. Keadle phases are: Phase 0: Mechanical signal testing, Phase I: Laboratory calibration using start/stop timing, Phase II: Laboratory or semi–free-living validation, including activity transitions, Phase III: Free-living validation, and Phase IV: Implementation in applied studies. This review included studies from Phases I–IV. Only articles published in or translated into English were eligible.

#### Exclusion criteria.

Studies were excluded if they did not incorporate a wearable sensor, focused solely on method development without empirical testing, investigated infant positioning during sleep, used wearables designed for non-motor parameters (such as sleep, physiological monitoring, or seizure detection), or were qualitative in nature.

### Study selection

After completion of the search, all identified articles were uploaded into Rayyan (Rayyan Systems, Cambridge, MA, USA), which is a reference manager platform designed to facilitate collaborative literature reviews. Duplicate records were removed prior to review. Four independent reviewers conducted titles and abstract screens to assess eligibility based on the inclusion criteria. Full texts of the included articles were then reviewed by two sets of two independent reviewers, each responsible for half of the articles, to determine final eligibility. Any disagreements regarding inclusion were resolved by a third independent reviewer. The results of the screening process are summarized in a PRISMA flow diagram.

### Data extraction and synthesis

Data were extracted from each article retained in accordance with Arksey and O’Malley framework for scoping reviews [10]. Two sets of two independent reviewers extracted data from the studies. A structured data extraction table was created to capture key study details such as study objective, study design, population type and age range, motor metrics assessed, sensor characteristics and data processing methods, methods used to evaluate accuracy, validity, or reliability of the device, as well as application of sensors for motor behavior assessment. Extracted data were synthesized descriptively and organized using Keadle’s five-phase validation framework to classify studies according to their emphasis on technical validation (Phases I–III) or applied implementation in real-world contexts (Phase IV). Results were summarized across phases to characterize patterns in validation approaches, analytic methods, and reported applications of wearable sensors for infant motor assessment, with the aim of mapping the current state of evidence rather than evaluating comparative effectiveness.

## Results

A total of 1,070 articles were identified initially. After removal of 102 duplicates, 968 unique records were screened. Following title and abstract screening based on the inclusion and exclusion criteria, 70 articles were retained for full-text review. Of these, 29 articles were excluded for not meeting the protocol inclusion criteria, resulting in 41 articles included in the final data extraction (Fig 1).

**Fig 1:**
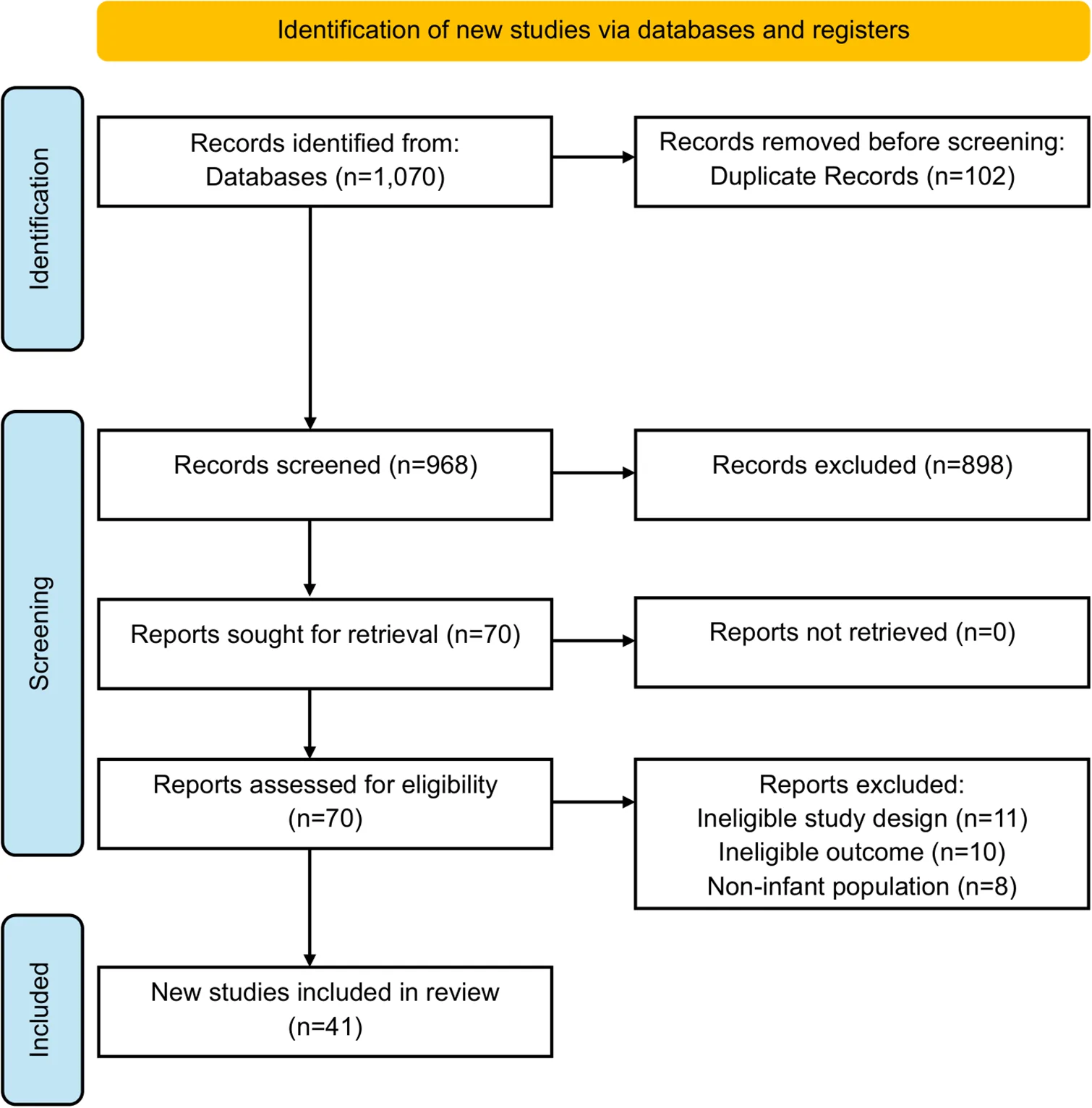
PRISMA flow diagram.

### Geographic locations and study settings

Study locations were determined by the author's affiliation when it was not explicitly stated in the article. Out of the 41 studies, 20 studies were conducted in North America (19 in the USA [11–29] and one in Canada [30]), 11 in Europe [31–41], four in Asia [42–45], two in South America [46,47], two in Australia [48,49], and two in Central America [50,51]. Data were most frequently collected in the home environment (n = 23) [12–14,16–31,35,39,46,47], followed by laboratory settings (n = 6) [15,32,36,37,40]. Some studies had mixed environments including “home and clinic” or “home and lab” (n = 3) [38,50,51]. Less common locations include clinics (n = 2) [33,40,44], NICU (n = 2) [34,48], childcare (n = 1) [45], and a health service group (n = 1) [49]. Two studies did not specify the data collection setting [42,43].

### Description of study participants

The included studies focused primarily on infants at or below 12 months of age, with enrollment age starting at birth (for full-term infants) and 28 weeks postmenstrual age (for preterm infants). Out of the 41 studies, 15 (36.6%) included only infants with typical development [11,15,17,18,23,27,30,32,33,40,42,45,49–51]. Of these, nine stated including infants born full term [11,17,18,30,33,40,42,50,51]. Twenty-two (53.7%) studies included infants at-risk for developmental disabilities or infants with diagnosed conditions. Of these 22 studies, six examined infants identified as at-risk for developmental disabilities [21,22,34,39,41,48]. At-risk was defined as either low birth weight, prematurity, medical complications requiring neonatal intensive care, early indicators of unilateral spastic cerebral palsy, high familial likelihood of autism spectrum disorder or severe brain injury diagnosed in the perinatal period. One study focused on infants with confirmed congenital conditions, specifically spinal muscular atrophy [25]. Fifteen studies enrolled mixed cohorts, where seven studies included both infants born preterm and infants that were typically developing [13,19,24,31,35,46,47], four studies included both typically developing infants and infants classified as at-risk for developmental delay [12,14,26,28], two studies included infants with typical development and infants with Down syndrome [16,20], one study involved both infants with typical development and those with motor disabilities (unspecified) [38], and one other study included typically developing infants and infants with a high familial likelihood of autism spectrum disorder [29]. Finally, four studies referred to participants as “infants” without additional information about developmental status [36,37,43,44]. Detailed study characteristics are presented in **Table 1**.

**Table 1:** Characteristics of included studies.

Study	Study Design	Study Location	Setting	Sample	Age range during study
(Ricardo et.al.,2019) [46]	Cross-sectional	Pelotas, Brazil	Home	2,974 infants (2,532 full-term; 442 preterm)	12 months
(Patel et.al., 2019) [11]	Cross-sectional	East Lansing, MI, USA	Lab	7 TD, full-term infants with caregivers	6-22 weeks
(Raza et.al., 2019) [31]	Cross-sectional	Wales, UK	Home	141 full-term and preterm infants with caregivers	12 months
(Abrishami et.al., 2019) [12]	Longitudinal	USA	Home	12 TD and 24 at-risk infants (preterm or perinatal complications)	TD: 1–8 months; At-risk: 2–15 months (adjusted)
(Fry et.al., 2019) [13]	Cross-sectional	Atlanta, GA, USA	Home	6 infants (3 full-term; 3 preterm)	Full-term: 2–8 months Preterm: 0.5–8 months adjusted
(Fitter et.al.,2019) [15]	Cross-sectional	Los Angeles, CA, USA	Lab	12 TD infants	6-8 months
(Hauck et.al., 2020) [16]	Longitudinal	Michigan, USA	Home	35 full-term infants (26 TD; 9 Down syndrome)	1-18 months
(Benjamin-Neelon et.al., 2020) [17]	Longitudinal	Southeastern, USA	Home	506 TD, full-term infants	3-12 months
(Fujii et.al., 2020) [42]	Case Study	Kanagawa and Tokyo, Japan	Home	1 TD infant	77 days
(Fitter et.al., 2020) [14]	Longitudinal	Portland, OR and Los Angeles, CA	Home	12 TD, full-term; and 24 at-risk infants	1-20 Months
(Trujillo-Priego et.al., 2020) [18]	Cross-sectional	California, USA	Home	10 TD, full-term infants	31–140 days
(Airaksinen et.al., 2020) [32]	Cross-sectional	Finland, Italy, Germany	Lab	24 TD infants	4.5-7.7 months
(Jun and Choi, 2020) [43]	Cross-sectional	Incheon, Korea	Unspecified	10 infants	2 days-12 months
(Hewitt et.al., 2020) [49]	Randomized Control Trial	Australia	Local area health services group	35 TD infants	Mean 5.9 months (SD 2.8 weeks)
(Whitehead et.al., 2020) [33]	Cross-sectional	London, England	Postnatal ward	11 TD, full-term infants	0.5-7 days
(Greenspan, et.al.,2021) [19]	Cross-sectional	Delaware, USA	Home	23 TD infants: 7 full-term infants in development group;13 full-term and 3 preterm infants in device-testing group	Development: mean 3.6 ± 1.2 months; Device Testing: mean 3.1 ± 1.1 months
(Fontana et.al., 2021) [34]	Cross-sectional	Italy	NICU	68 infants at risk (preterm, VLBW, severe brain injury)	Mean PMA 42.1 ± 2.4 weeks
(Khasgiwale et.al., 2021) [20]	Longitudinal	Pacific Northwest & Southern California, USA	Home	18 infants (9 Down syndrome; 9 TD)	Down syndrome: 3–5 months at onset; TD: 4–7 months
(Wilson et.al., 2021) [21]	Longitudinal	California, USA	Home	5 infants at risk for ASD	3-12 months
(Rosales et.al., 2022) [22]	Prospective cohort longitudinal	Los Angeles, California, USA	Home	12 infants at risk (preterm, LBW, NICU stay)	3-9 months (adjusted)
(Jardine et.al., 2022) [48]	Prospective observational	Queensland, Australia	NICU	59 extremely preterm/ or low birth weight infants	28–32 weeks PMA
(Shull et.al., 2022) [23]	Cross-sectional	Southeastern Region, USA	Home	143 TD infants	6-7 months
(Zhang et.al., 2022) [30]	Cross-sectional	Canada	Home	29 TD, full-term infants	2-6 months
(Airaksinen et.al., 2022) [35]	Cross-sectional	Helsinki, Finland	Home or Home-Like Environment	59 infants (38 low-risk term; 10 mild prenatal asphyxia; 11 preterm)	5-19 months
(Laudańska et.al.,2022a) [36]	Longitudinal	Poland	Lab	26 infants with their mothers	4-12 months
(Laudańska et.al.,2022b) [37]	Longitudinal	Poland	Lab	40 infants	4-12 months
(Fry-Hilderbrand et.al., 2022) [24]	Cross-sectional	USA	Home	23 infants (15 full-term; 8 preterm)	1-10 months
(McIntyre et.al, 2023) [25]	Longitudinal case report	Los Angeles, USA	Home	1 infant with SMA	7 months 23 days to 21 months 10 days
(de Andrade Leão et.al., 2023) [47]	Prospective, longitudinal	Pelotas, Brazil	Home	4275 Full-term & preterm infants	Birth–48 months
(Deng et.al., 2023) [26]	Cross-sectional	Los Angeles, USA	Home	11 TD and 16 at-risk infants	TD: 1–10 months; At-risk: 2–14 months
(Vaaras et.al., 2023) [38]	Cross-sectional	Finland, Italy, Germany	Home and lab	DS1: 62 infants (3 with motor disabilities); DS2: 41 infants (3 with motor disabilities)	4-20 months
(Franchi de’Cavalieri et.al., 2023) [39]	Longitudinal	Tuscany, Italy	Home	17 infants with perinatal brain injury (8 preterm, 9 term)	3.12 to 13.8 months (adjusted)
(Koziol et.al., 2023) [40]	Cross-sectional	Poland	Lab	20 TD full-term infants	3.9 to 4.9 months
(Verhage et.al,2024) [41]	Prospective Longitudinal	University Medical Center Utrecht, The Netherlands	Clinic	50 infants with unilateral perinatal brain injury at high risk for USCP	3-12 months
(Franchak et.al., 2024) [27]	Longitudinal	University of California Riverside, Riverside, CA	Home	22 TD infants	Two groups: 3.8–7.2 months and 10.7–14.2 months
(Ghazi et.al., 2024) [28]	Prospective Longitudinal	Los Angeles CA, USA	Home	10 infants (6 TD; 4 at-risk)	31 to 433 days (adjusted)
(Bao et.al., 2024) [44]	Cross-sectional	Quwo, Linfen in China	Clinic	23 infants	4-21 weeks
(Oh et.al., 2024a) [51]	Longitudinal	Guatemala	Home or Clinic	41 TD, full-term infants	0–6 months
(Oh et.al., 2024b) [50]	Longitudinal	Guatemala	Home or Clinic	40 TD full-term infants	0-6 months
(Iverson et.al., 2024) [29]	Longitudinal	University of Pittsburgh, Pittsburgh, PA, USA	Home	44 infants (24 with TD and 20 at risk for ASD)	2.5 - 36 months
(Onodera et.al.,2024) [45]	Cross-sectional	Japan	Childcare	24 infants	6-24 months

TD = typical development, SD = standard deviation, NICU = neonatal intensive care unit, ASD = autism spectrum disorder; SMA = spinal muscular atrophy; VLBW = very low birth weight; LBW = low birth weight, PMA = post-menstrual age, USCP = unilateral spastic cerebral palsy, DS1 = large unlabeled dataset of MAIJU data, DS2 = smaller labeled dataset of MAIJU data

*Studies that enrolled infants over 12 months of age were included if at least 50% of participants fell within the 0–12-month range or if data for this subgroup were reported separately

### Sensors

Of the 41 total articles,15 utilized standalone accelerometers [11,16,17,19,30,31,34,39,41,43,45–49] while 24 utilized accelerometers as part of an inertial measurement unit (IMU) [12–15,18,20–22,24–29,32,33,35–38,40,44,50,51]. One study utilized both an accelerometer and an IMU [23] while another used an accelerometer and a strain sensor [42]. Sensors were either embedded in garments (e.g., sensor suits, jumpsuits, and the Get Around Garment) or attached directly to specific body locations with a pouch or strap. Sampling frequencies ranged from 1 Hz to 150 Hz, reflecting differences in the temporal resolution needed to capture the targeted movement characteristics. Between 1–6 sensors were applied to infants across the studies. Sensors were most frequently placed on the ankles, followed by the legs, arms or wrists, and trunk or feet. Less common placement sites included the chest, hip, waist, hands, and forehead.

### Study aim 1: Validation studies (Keadle phases I-III)

Detailed study-level information, including sensor characteristics, outcome definitions, processing methods, reference standards, and validation metrics, is provided in Table 2.

**Table 2:** Infant wearable sensor systems in the validation phase (Keadle Phase I-III).

Study	Study Aim	Type of sensor	Sample rate	Sensor location (n)	Outcome Category and Metrics	Validation Category and Metrics	Phase	Reference Standard	Processing Method
(Patel et.al., 2019) [11]	Investigate impact of physical contact and infant position on spontaneous movements using a two-body networked sensor system	Custom 3-axis accelerom-eters	NS	Right upper arm (1)	**Movement** Infant vs. caregiver-generated movement during holding (Caregiver contact) Active duration of arm movements, acceleration peaks per unit time, dimensionless jerk	**Signal fidelity** Hold/No-Hold random forest classifier accuracy = 86.7% Kalman filter performance: 92.2% reduction in non-infant signal power tested using inanimate surrogate.	Phase I	Video recording; known zero-motion from inanimate infant; caregiver accelerometer	Random forest classifier for activity categorization; Kalman-filter algorithm to isolate infant-generated movement
(Fry et.al., 2019) [13]	Develop system for early detection of motor delay and cerebral palsy from spontaneous kicking in natural settings	Bluetooth infant suit with six MbientLabMetawearC6-axis IMUs	100 Hz	Thighs, shins, feet (6)	**Movement** no motion, unilateral, bilateral	**Classification Performance** KNN ACC = 81.2–83.7% (across sensor combinations);	Phase II	Time-stamped video recordings	Sliding window + SHOD to generate features; classification via thresholding, KNN, GMM models.
(Fujii et.al., 2020) [42]	Test whether strain-sensor suit can detect limb movement while minimizing caregiver movement artifacts	Wearable strain sensor suit: Xenoma “e-skin coo” strain sensors; 3-axis accelerometer	32 Hz	Strain sensors on arms/leg (3); accelerometer on trunk (1)	**Movement** Infant limb movement vs. caregiver-associated movement	**Signal fidelity** Low correlations (r < 0.5) between strain-sensor signals and both infant- and caregiver-worn accelerometers suggested that the suit captured a distinct infant limb-movement signal, whereas high infant–caregiver accelerometer correlations (r > 0.5) reflected shared motion during holding. Strain-sensor suit shows potential use for measuring infant limb movement in caregiver–infant interactions.	Phase I	Comparisons made to accelerometers	Accelerometer data converted to m/s²; strain sensor data converted to kΩ, filtered, differentiated; RMS used for activity quantification
(Airaksinen et.al., 2020) [32]	Develop multisensor smart jumpsuit for accelerometer and gyroscope recording of infant movements	**Smart Jumpsuit**: Suunto Movesense IMUs	52 Hz	Upper arms, upper legs (4)	**Posture:** prone, supine, side left (L), side right (R), and crawl **Movement:** still, turn left (L), turn right (R), pivot left (L), pivot right (R), crawl proto, crawl commando and crawl 4 limbs	**Classification Performance** Posture ACC = 98.2%, UAP = 93.3%, UAR = 94.1%, UAF = 93.7%. Movement ACC = 81.7%, UAP = 75.4%, UAR = 71.9%, UAF = 72.7%	Phase II	Video Recording	Windowing with overlap; SVM and deep CNN classifiers
(Jun and Choi, 2020) [43]	Classify four infant activity types	Chois Technology device with 3-axis accelerometer and thermometer	40 Hz	Chest (1)	**Movement** sleeping, strong movement, weak movement, external force	**Classification Performance** Avg precision = 0.98; recall = 0.93; specificity = 0.98; balanced ACC = 96%; F1 = 0.95	Phase II	Video Recordings	Mean-subtracted segments; cumulative absolute difference; unsupervised deep model
(Greenspan, et.al.,2021) [19]	Develop and validate the Get Around Garment (GG) for infant body-position classification	**Get Around Garment**: ADXL335 triaxial accelerometer	1 Hz	Right trunk at ASIS level (1)	**Posture** supine, reclined, inclined, prone	**Comparison with reference standard** Second-by-Second agreement for each position: Overall Weighted κ = 0.837; 95% confidence interval = .833−.841 (interpreted as “excellent or almost perfect”) Correlation for cumulative time in each posture r=0.916, p<0.0001 (interpreted as “very strong”)	Phase II	Video recording of infant body positions	Acceleration converted to pitch angle; classification via custom LabVIEW algorithm based on pitch angle data
(Fontana et.al., 2021) [34]	Develop automatic quantitative index to identify abnormal limb movements at term-equivalent age	LIS3LV02DL accelerometer	150 Hz	Wrists, ankles (4)	**Movement** KC Index: limb-specific kurtosis + inter-limb jerk cross-correlation	**Discrimination of clinically relevant movement patterns** Specificity = 0.86; sensitivity = 0.88; AUC = 0.89 (95% CI 0.81–0.97); OR (GMA vs KC- Index) = 1.058	Phase II	GMA video assessments	Signals were high-pass filtered to remove gravitational components. The KC-index was computed by combining the normalized kurtosis of the first principal component and the normalized cross-correlation of the jerk. Discriminatory power was assessed using logistic regression and ROC curve analysis
(Jardine et.al., 2022) [48]	Determine whether quantitative accelerometry correlates with GMA in preterm infants (28 and 32 PMA)	Commercial tri-axial accelerometers	80 Hz	Dorsum of hands and feet (4)	**Movement** Spontaneous movements Max acceleration/velocity, cross-correlation, skewness, deviation from moving average	**Comparison with reference standard** 28-Weeks PMA: 7 of 46 (15.2%) accelerometer parameters differed significantly (P < 0.05) between infants with normal and abnormal GMs 32-Weeks PMA: 19 of 46 (41.3%) accelerometer parameters differed significantly (P < 0.05), demonstrating stronger correlation and sensitivity at later gestation	Phase III	GMA from video (two raters + adjudication)	Forty-six parameters computed per Meinecke et al.: third-party analytical pipeline
(Airaksinen et.al., 2022) [35]	Construct and validate scalable method for measuring spontaneous motor abilities from supine to walking	**MAIJU Wearable Sensor Suit:** Movesense IMUs (accelerometer + gyroscope)	52 Hz	Proximal limbs via MAIJU suit pockets (4)	**Posture:** Prone, Supine, Side Left, Side Right, Crawl, Sitting, Standing **Movement:** Still, Roll Left, Roll Right, Pivot Left, Pivot Right, Proto, Elementary, Fluent, Transition	**Classification Performance** Overall Posture ACC = 95.8% UWF1 = 92.4% Overall Movement ACC = 82.5% UWF1 = 74.8% **Comparison with reference standard (human)** For time spent in each posture and movement Posture Prone, r = 0.99 Supine, r = 0.99 Side, r = 0.97 Crawl, r = 0.98 Sitting, r = 0.99 Standing, r = 0.99 Movement Still, r = 0.95 Roll, r = 0.98 Pivot, r = 0.96 Proto, r = 0.83 Elementary, r = 0.91 Fluent, r = 0.96 Transition, r = 0.97	Phase II	Video Recording	Gyroscope bias removal, smoothing, segmentation; CNN classifier; binary filter; MAE + Spearman correlation evaluation
(Vaaras et.al., 2023) [38]	Test whether self-supervised pre-training improves infant-movement classification	**MAIJU Wearable Sensor Suit:** (accelerometer + gyroscope)	52 Hz	Four limb sensors (4)	**Movement**: Still, Rol, Pivot, Proto, Elementary, Fluent	**Classification Performance** UAF1 = 82.1%; precision = 78.3%; recall = 86.9%	Phase II	DS2-video recordings	Preprocessing + windowing; pre-training on unlabeled data; fine-tuning on labeled dataset
(Verhage et.al, 2024) [41]	Detect asymmetric upper limb movement in infants at risk for unilateral spastic cerebral palsy	ActicalVR accelerometers	32 Hz	Wrists (2)	**Movement** Upper-limb acceleration; asymmetry index (AI)	**Comparison with reference standard:** Age-specific person r between AI _ACC_ and AI_HAI_: 3–5 months, r = 0.61, 5–7.5 months, r = 0.81, 7.5–12 months, r = 0.84 **Discrimination of clinically relevant movement patterns** AUC: 3-5 months, 0.96; 5-7.5 months 0.93, 7.5-12 months 0.88	Phase II	HAI from video by therapist	Actical 3-axis counts; visual inspection for signal quality
(Franchak et.al., 2024) [27]	Validate home-based body-position classification and assess generalization	**Sensor Garment:** MC10 BioStamp IMUs	62.5 Hz	Thighs and ankles (4)	**Posture** supine, prone, sitting, upright, held	**Classification Performance** Assessed semi-supervised accuracy Group model (aggregated data from all infants to create a single body position classifier): ACC = 84.6%, κ = 0.746 (interpreted as “Substantial”), Sensitivity = 0.825, PPV = 0.819. Individual model (trained on one participant’s data to predict their later behavior): ACC = 91.6%, κ = 0.821 ((interpreted as “Almost Perfect”), sensitivity = 0.841, PPV = 0.899; **Comparison with reference standard** Assessed long-term unsupervised recording for time spent in each posture Group model r = 0.80 Individual model r = 0.91	Phase III	Video Recording	Video–IMU synchronization via drop event; R-based timestamp matching; Random Forest group/individual models
(Ghazi et.al., 2024) [28]	Establish accelerometer thresholds to classify PA intensity	APDM Opal sensor (IMU)	20 Hz	Ankles (2)	**Movement** Activity counts; intensity thresholds – Sedentary / light / moderate-to-vigorous (MV) activity	**Classification Performance** TPR/TNR by activity intensity Sedentary: 76.4% / 90.0%; Light: 51.5% / 84.4%; MV: 68.3% / 78.1%. Predicted activity proportions closely matched video: Sedentary 36.9% vs. 40.6%, Light 22.3% vs. 18.6%, MV 40.8% vs. 40.8%.	Phase I	Video recording	Gravity-removed acceleration scaled by leg length; AUC and jerk computed over intervals; threshold optimization
(Bao et.al., 2024) [44]	Validate IMU system for GMA scoring and developmental-risk detection in low-resource setting	Soft wireless IMUs (SSN-SWDs)	30 Hz	Ankles, wrists, forehead (5)	**Movement** GMA categories (normal/low/high risk) classified using time-, frequency-, and entropy-domain features	**Discrimination of clinically relevant movement patterns** Binary risk model accuracy = 99.9%; logistic regression accuracy = 95.3%	Phase I	Clinical evaluation + video; commercial IMU	Missing-data interpolation; 14-Hz Butterworth filter; 120-s windows; 36 features; t-tests + SMOTE; LR and CNN + additional classifiers
(Onodera et.al.,2024) [45]	Recognize multilabeled infant activities using low-sampling-rate chest accelerometer	ICUCO triaxial accelerometer	7 Hz	Chest (1)	**Movement and Posture** Up to 14 simultaneous or daily actions such as sleeping, crawling, being held horizontally, walking, or standing	**Classification Performance** LP + LightGBM model: precision = 0.88; recall = 0.89; F1 = 0.88 (LOOCV)	Phase I	Time-stamped video recordings	Sliding-window feature extraction, z-normalization; multilabel LP + LightGBM classifier

NS = not specified, IMU = inertial measurement unit, KNN = k-nearest neighbors, R² = coefficient of determination, SHOD = stance hypothesis optimal detector, GMM = Gaussian mixture model, PA = physical activity, r = Pearson’s correlation coefficient, kΩ = kilo-ohms, RMS = root mean square, ACC = accuracy, UAP = unweighted average precision, UAR = unweighted average recall, UAF = unweighted average F1-score. F1 scores are machine-learning evaluation metrics that reflect how well a classifier correctly identifies movements while minimizing missed detections and false alarms, particularly when some movement classes occur infrequently, KC = Kurtosis–Correlation Index,SVM = support vector machine, CNN = convolutional neural network, Avg = average, ASIS = anterior superior iliac spine, κ = kappa coefficient, AUC = area under the curve, CI = confidence interval, OR= odds ratio, GMA = general movement assessment, PMA = post-menstrual age, BIMS = BABA Infant Motor Score, UAF1 = unweighted average F1, DS2 = smaller labeled dataset of MAIJU data, AI_ACC_ = asymmetry index for the accelerometer, AI_HAI_ = asymmetry index for the Hand Assessment for Infants, HAI = Hand Assessment for Infants, MAE = mean absolute error, PPV = positive predictive value, SD = standard deviation, TPR = true positive rate, TNR = true negative rate, MV = moderate-to-vigorous, SSN-SWD = Sparse Sensor Network – Soft Wireless IMU Devices, SMOTE = synthetic minority oversampling technique, LR = logistic regression, LP = label powerset, LightGBM = light gradient boosting machine, LOOCV = leave-one-out cross-validation

#### Outcome category distribution.

Out of the 41 studies included in this review, 15 (36.6%) were validation studies. The included studies used accelerometer- or inertial measurement unit–based systems to quantify infant posture (n = 2) [19,27], movement (n = 10) [11,13,28,34,38,41–44,48] or both (n = 3) [32,35,45]. Posture outcomes included supine, prone, side-lying, reclined, inclined, sitting, standing, upright, and being held. Movement outcomes ranged from broad states, including stillness, sleep, activity intensity, and externally imposed movement, to more specific motor behaviors, including unilateral or bilateral kicking, rolling, pivoting, crawling, proto, elementary, and fluent movement. Several studies also quantified clinically oriented movement constructs, including general movements, upper-limb asymmetry, and developmental-risk classification.

#### Validation category distribution.

Validation outcomes were grouped according to the question each metric addressed: (1) classification performance; (2) comparison with a reference standard; (3) discrimination of clinically relevant movement patterns; and (4) signal fidelity. These categories were not mutually exclusive; several studies reported more than one type of validation evidence.

A visual summary of classification performance and reference-standard validity across Keadle Phase I–III studies is presented in Fig 2.

**Fig 2:**
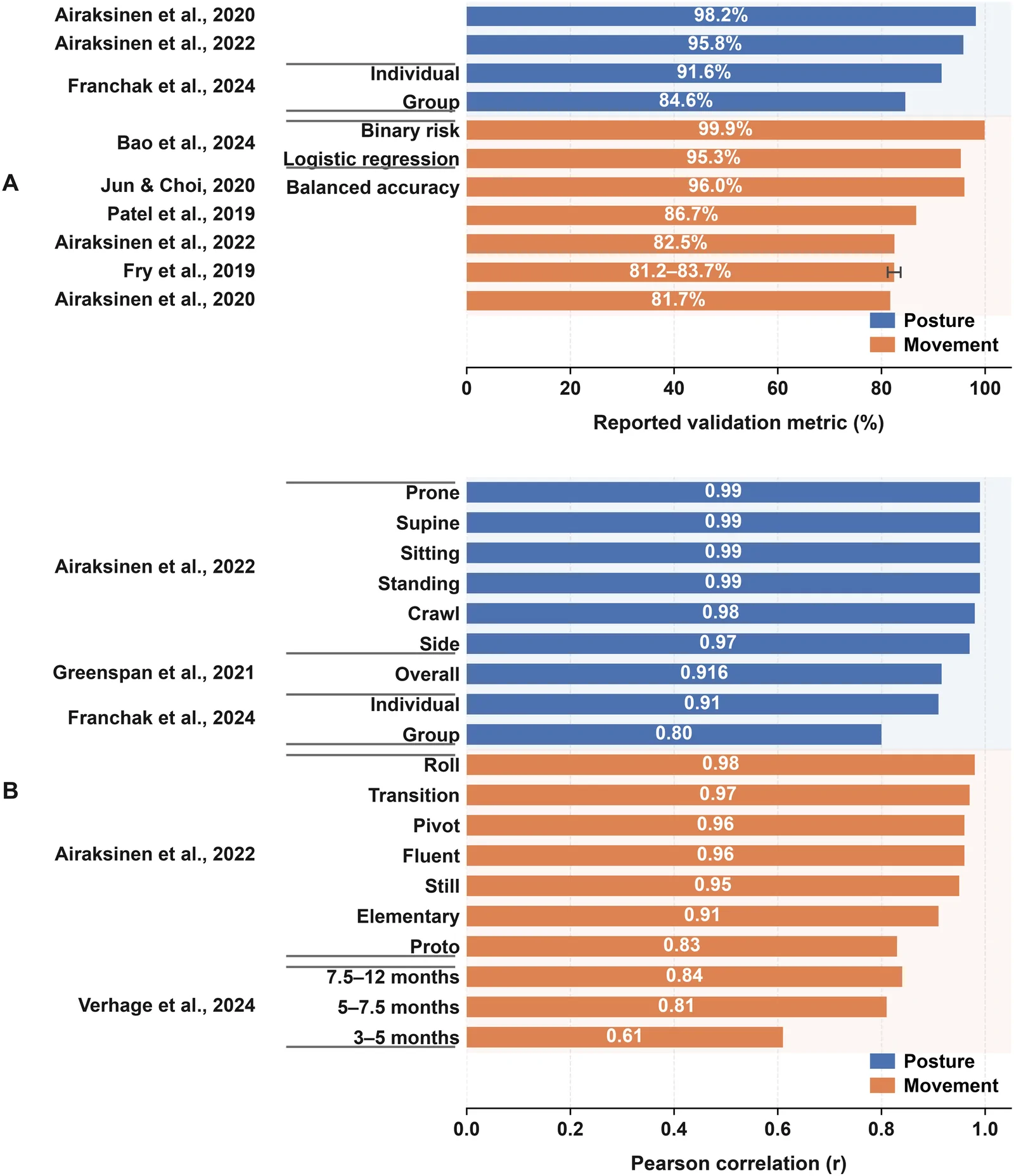
Classification performance and correlation with reference standards for wearable sensor systems in infant validation studies (Keadle Phases I–III). A) Reported classification accuracy or balanced accuracy (%) across posture, movement, combined posture-and-movement. Studies reporting F1, UAF1, TPR/TNR, or other non-accuracy metrics are excluded (B) Pearson correlations between wearable-derived measures and video-, human-, or clinician-derived reference standards. Values are presented as reported by each study and are not directly comparable across differing behavioral outcomes, sensor configurations, reference standards, or validation designs.

#### Classification performance.

Nine studies reported classification performance, indicating how accurately an algorithm assigned posture or movement labels using accuracy, precision, recall, specificity, F1 score, balanced accuracy, kappa, sensitivity, positive predictive value, or class-specific true-positive and true-negative rates [13,27,28,32,35,38,43–45]. Performance was generally higher for posture than movement when both outcomes were evaluated within the same study. In the two MAIJU suit studies, posture classification accuracy was 98.2% and 95.8%, compared with 81.7% and 82.5% for movement classification; posture F1 scores were also higher than movement F1 scores (93.7% vs. 72.7% and 92.4% vs. 74.8%, respectively) [32,35]. Posture-classification performance also varied by model design, with one study reporting that individualized model achieved higher accuracy than a group model (91.6% vs. 84.6%) [27]. Movement-only classification performance was more variable than posture classification and appeared to depend on the specificity of the target behavior. Studies distinguishing several movement types generally reported accuracy or F1 values in the low 80% range, with category-specific performance varying substantially. In contrast, studies using broader activity labels or combined posture-and-movement categories reported higher aggregate performance, including balanced accuracy of 96% for four activity states [43] and an F1 score of 0.88 for multilabel daily-activity classification [45].

#### Reference-standard comparison.

Comparison with a reference standard, indicating the agreement or association between sensor-derived estimates and video-, human- or clinician-derived measures was reported in seven studies [19,27,28,32,35,41,48]. Posture estimates generally demonstrated strong correspondence with video-derived reference standards. Cumulative posture-duration correlations ranged from r = 0.80 to r = 0.99 [27,35], with the strongest values reported for the MAIJU posture categories (r = 0.97–0.99) [35]. Greenspan et al. similarly reported strong second-by-second posture agreement (k = 0.837) and a correlation of r = 0.916 for cumulative posture time [19]. These results indicate that wearable systems can provide valid estimates of time spent in relatively stable postures. Movement estimates showed greater variability in correspondence with reference standards. In the MAIJU study, correlations for cumulative movement duration ranged from r = 0.83 for proto movement to r = 0.98 for rolling, with stillness, pivoting, elementary movement, fluent movement, and transitions ranging from r = 0.91 to r = 0.97 [35]. For clinically oriented movement measures, associations with reference standards varied by developmental age. Accelerometer-derived upper-limb asymmetry showed correlations with therapist-rated asymmetry ranging from r = 0.61 at 3–5 months to r = 0.84 at 7.5–12 months [41]. In preterm infants, a greater proportion of accelerometer-derived movement parameters differentiated normal froms abnormal general movements at 32 weeks than at 28 weeks postmenstrual age (41.3% vs. 15.2%) [48].

#### Clinical discrimination.

Discrimination of clinically relevant movement patterns or developmental risk, indicating how well sensor-derived features distinguished clinically defined motor patterns, developmental risk, or impairment, was evaluated in four studies [34,41,44,48]. The Kurtosis–Correlation Index achieved sensitivity of 0.88, specificity of 0.86, and an Area Under Curve of 0.89 for differentiating clinically relevant general-movement patterns at term-equivalent age [34]. Accelerometer-derived upper-limb asymmetry discriminated infants at risk for unilateral spastic cerebral palsy, with Area Under Curve values of 0.96, 0.93, and 0.88 across the 3–5-, 5–7.5-, and 7.5–12-month age periods, respectively [41]. General-movement risk classification achieved 99.9% accuracy for a binary model and 95.3% accuracy for logistic regression [44].

#### Signal fidelity.

Signal fidelity was evaluated in two early-phase studies [11,42]. These studies addressed whether the wearable signal accurately represents the infant’s own movement rather than artifact, sensor noise, garment movement, caregiver handling, or shared caregiver–infant motion. Patel et al. demonstrated that a two-body sensor system could identify whether an infant was being held and reduce caregiver-related contamination in the infant sensor signal [11]. Similarly, Fujii et al. found that strain-sensor signals were weakly correlated with both infant- and caregiver-worn accelerometers (r < 0.50), whereas infant–caregiver accelerometer signals were more strongly correlated during holding (r > 0.50) [42]. Together, these findings provide preliminary evidence that signal-processing approaches and strain-sensor garments may help distinguish infant limb movement from shared caregiver–infant motion.

### Study aim 2: Application studies (Keadle Phase IV)

Of the 41 studies included, 26 (63.4%) were application-focused Keadle Phase IV studies [9] that implemented previously validated sensors and algorithms to obtain objective measurements of infant motor activity. Table 3 outlines results for application studies.

**Table 3:** Application of wearable sensors (Keadle Phase IV).

Study	Study Aim	Type of sensors	Sample Rate	Sensor Location (n)	Outcomes	Processing Method
(Ricardo et.al.,2019) [46]	Describe objectively measured overall PA and its correlates in one-year olds	ActiGraph, model wGT3X-BT accelerometer	60 Hz, 5 sec epoch	Left wrist (1)	**PA**: Average daily acceleration	Raw data were processed using the GGIR, R-package, which included auto-calibration, detection and imputation of non-wear periods, and calculation of the ENMO metric.
(Raza et.al., 2019) [31]	To explore factors associated with PA levels of one-year olds	GENEActiv triaxial accelerometer	100 Hz	Ankle (1)	**PA**: signal vector magnitude (SVM) and the movement intensity (MI) was derived from these SVMs, with the unit for MI being g (1g = 9.81 m·s − 2)	PA intensity was quantified using gravity-subtracted SVMs.
(Fitter et.al.,2019) [15]	To explore whether infants increase leg movements in response to contingent humanoid robot feedback to establish automated early interventions for developmental delays	APDM Opal Sensor (IMU)	128 Hz	Arms and legs (4)	**Leg movement characteristics**: Frequency of movements (moves/minute) and Ball kicks/minute	Real-time limb acceleration was calculated via a quaternion filter, with robot rewards triggered whenever the infant's leg movement exceeded threshold
(Abrishami et.al., 2019) [12]	To differentiate infants with typical development from those at risk by analyzing statistical features of full-day leg movements and evaluating their relationship with developmental outcomes at 24 months.	APDM Opal Sensor (IMU)	20 Hz	Ankles (2)	**Leg movement characteristics**: Duration of a movement, peak acceleration, and average acceleration during leg movement	Individual leg movements were identified from raw acceleration signals and characterized by kinematic features, with their full-day distributions summarized via statistical moments for model input.
(Hauck et.al., 2020) [16]	To compare the trajectories and interplay of PA and motor skill development between infants with Down syndrome and typically developing infants during their first 18 months	Actical accelerometer	Sampling frequency not reported but activity was recorded using 15-second epochs	Right ankle (1)	**PA**: Average activity counts per minute over the 24-hour period	A rigorous manual validation process was employe to isolate infant-generated physical activity from passive motion
(Benjamin-Neelon et.al., 2020) [17]	To assess accelerometer measured PA and its associations to adiposity over four equally spaced visits in the first year of life	wActiSleep+ monitor	30 Hz	Right Ankle (1)	**PA**: Activity counts	Acceleration data were aggregated into 1-minute epochs, log-transformed to mitigate skewness, and standardized to quantify total daily physical activity volume
(Trujillo-Priego et.al., 2020) [18]	To characterize the intensity of infant leg movements in the 20 minutes before and after nap periods using wearable sensors	APDM Opal Sensor (IMU)	20 Hz	Ankles (2)	**Leg movement characteristics**: intensity	Sleep events were detected by calculating the acceleration norm, followed by low-pass filtering and full-wave rectification. Leg movement intensity was quantified as the AUC
(Hewitt et.al., 2020) [49]	To determine the feasibility, acceptability, and potential efficacy of a group intervention to promote tummy time	GENEActiv Triaxial Accelerometer	30 Hz	Right hip (1)	**Intervention responsiveness** (Tummy time): minutes per day	Accelerometer wear/non-wear time was classified using temperature and z-axis data. A Threshold-based algorithm quantified tummy time position and its duration
(Fitter et.al., 2020) [14]	To use day-long kinematic data collected from wearable inertial sensors to classify infants as typically developing or at risk for developmental delay. Also aimed to predict future outcomes for AR infants	APDM Opal Sensor (IMU)	20 Hz	Ankles (2)	**Leg movement characteristics:** Movement duration. motion acceleration, average peak acceleration, unilateral movement rates, bilateral asynchronous movement rates, overall leg movement rates, bilateral synchronous movement rates	A baseline of 23 features was expanded with motion-rate ratios and subsequently reduced via Lasso regression to eight critical predictors for classification.
(Whitehead et.al., 2020) [33]	To quantitatively characterize the long-range temporal organization of motor activity	APDM Opal Sensor (IMU)	Collected at 128 Hz and down sampled by a factor of 6	Legs (2); Arms and legs (4) for 7/11 infants	**Movement organization, complexity, and coordination:** Frequency of movements, proportion of bilateral movements, movement clusters (min, max, median, silhouette coefficient), spectral periodicity of clusters of movement, peaks in movement occurrence, peak acceleration	Movements were identified using adaptive, individual-level acceleration and angular velocity thresholds to exclude caregiver handling, with temporal patterns subsequently analyzed through cluster analysis and spectral density estimation.
(Khasgiwale et.al., 2021) [20]	To measure leg movement rates using sensors in infants with DS before and after an intervention to promote kicking	APDM Opal Sensor (IMU)	Data were collected at 128 samples per second for DS infants and then down sampled by factor of 6 to be similar to data collected at 20 samples per second from TD infants	Ankles (2)	**Intervention responsiveness:** Leg movement rate (movements per hour of awake time), Leg Movement Duration (seconds per movement), Average acceleration (m/sec^2^); and Peak acceleration (m/sec^2^)	MATLAB was used to analyze the sensor data. Data was down sampled from 128 to 20. Statistical analysis included descriptive statistics, ANOVA, and t-tests
(Wilson et.al., 2021) [21]	To use wearable sensor data to develop a motion complexity metric and determine if there is a correlation between lower motion complexity and ASD diagnosis	APDM Opal Sensor (IMU)	20 Hz	Ankles (2)	**Movement organization, complexity, and coordination**: Duration of movement, peak acceleration, and average acceleration of kicking. Used to derive a Motion Complexity Index	After z-score normalization, 5-minute active segments were analyzed via Fast Fourier Transform (FFT) to calculate a Motion Complexity Index based on the amplitude-weighted standard deviation of frequency components
(Shull et.al., 2022) [23]	To describe objectively measured PA using accelerometers in 6–7-month-old infants; compare PA levels by demographics and developmental factors; To identify the demographic and developmental factors that are correlates to PA	ActiGraph GT3X+ model (waist) and GT9X model (ankle)	80Hz	Right waist and right ankle (2)	**PA**: Average activity counts per hour	Acceleration data were aggregated and quantified as VM average counts per hour, following the exclusion of non-wear periods defined by 60 consecutive minutes of zero counts
(Rosales et.al., 2022) [22]	To quantify the impact of caregiver-led home intervention on leg movement rates in at-risk infants while evaluating protocol adherence and the influence of caregiver-child interaction quality	APDM Opal Sensor (IMU)	20 Hz	Ankles (2)	**Intervention responsiveness:** leg movement rate per hour	Leg movement rates were calculated by a MATLAB algorithm that identified discrete movements based on kinematic pauses or direction changes and was validated against video observation
(Zhang et.al., 2022) [30]	Examine the concurrent validity of infant tummy time questionnaire and time-use diary against the GENEActiv accelerometer	GENEActiv triaxial accelerometer	30 Hz	Right hip (1)	**PA** (Tummy time): minutes per day	Raw data was converted into 1-second epoch .csv files using GENEActiv software. A Threshold-based algoithm quantified tummy time position and its duration
(Laudańska et.al.,2022a) [36]	To investigate the changes in the complexity of limb movements across infancy in two tasks that differ in the level of structuring (rattle-shaking and free play)	Xsens MTw Awinda Wearable Motion Tracker	60 Hz	Arms and legs (4)	**Movement organization, complexity, and coordination:** Entropy, Recurrence Rate, and Mean Line	After interpolated Missing values, 3D acceleration reduced to a 1D magnitude, and z-scored t before MdRQA to extract limb coupling features.
(Laudańska et.al.,2022b) [37]	To study the developmental changes in motor coordination between arm movements during rattle-shaking	Xsens MTw Awinda Wearable Motion Tracker	60 Hz	Arms (2)	**Movement organization, complexity, and coordination:** Number of movements, frequency, rattling frequency, between-arm coherence	Missing samples were interpolated, and IMU data was reduced to a smoothed acceleration magnitude. Data was synchronized with video. Wavelet coherence between hands was computed. Episodes were classified
(Fry-Hilderbrand et.al., 2022) [24]	Assess infant motor development by extracting kinematic features from spontaneous kicking, identifying features that predict age, and establishing normative developmental values across ages, and compare preterm vs term values	MbientLab’s MetawearC wireless sensors	100 Hz	Thighs, shins, and feet (6)	**Leg movement characteristics:** An activity vector for each leg, frequency of activity, Interlimb coordination for bilateral activity (BiFi), unilateral left activity (ULFi), and unilateral right activity (URFi), infant's predominant leg, average and maximum active kicking and rest period duration, peak and average acceleration for infant's predominant leg, maximum joint excursion for each degree of freedom of the predominant leg	Gyroscope drift was mitigated via zero-velocity updates before segmenting data into 1-minute blocks to extract coordination vectors, kick-cycle kinematics, and PCA-based joint excursions.
(de Andrade Leão et.al., 2023) [47]	Examine the relationship between PA and growth indicators in children under five	ActiGraph wGT3X-BT accelerometers	Not specified	Left wrist (1)	**PA**: Mean acceleration per day (mg) + SD	Data were analyzed using the R-package GGIR. Overall PA was using ENMO (raw acceleration), a three-dimensional metric
(McIntyre et.al, 2023) [25]	Report the quantity and kinematic characteristics of leg movements in a symptomatic SMA type I child (2 copies of SMN2) treated with disease-modifying therapy	APDM Opal Sensor (IMU)	20 Hz	Ankles (2)	**Leg movement characteristics:** Movement rate (number of movements per hour awake across a full day), average duration, peak acceleration, and average acceleration	For each movement, duration, peak acceleration, and average acceleration are computed and averaged across all movements to summarize daily movement characteristics.
(Deng et.al., 2023) [26]	To apply a linear method, a nonlinear method, and barcoding analyses to compare full-day, in-home leg movement patterns in TD and AR infants using wearable sensors	APDM Opal Sensor (IMU)	20 Hz	Ankles (2)	**Leg movement characteristics:** Movement onset/offset, duration, peak acceleration, and peak velocity of each leg movement. Linear and non-linear variation Four dimensions for barcoding analysis (peak acceleration, peak angular velocity, duration, and move- ment type) to determine barcoding states Sample entropy	Awake times were analyzed. Movements were detected, and Sample Entropy extracted, then classified into 16 barcoding states by intensity, duration, and laterality
(Franchi de’Cavalieri et.al, 2023) [39]	Test if there is a significant relation between the accelerometer data during CT-R play and the scores of IMP and AIMS clinical assessment at baseline and test that clinical outcomes can be predicted from the quantitative accelerometric data combined with baseline clinical data	Axtivity AX3, Tri-axial Accelerometer	25 Hz	Wrist and trunk (3)	**Movement organization, complexity, and coordination**: AIX: SVM minus 1g; Asymmetry Index (AI); Movement Relative to Trunk (AI hand/trunk); Total Movement (TotalMovement); Relative Asymmetry Index (AI MP,LPtrunk) where MP = most preferred hand, LP = least preferred hand	Data processed using OmGui, MATLAB, and R. Calculated indexes include SVM and AIX. Statistical analysis employed Functional Data Analysis (FDA) and Functional Linear Models (FLM)
(Kozioł et.al., 2023) [40]	Quantify overflow and analyze coordination of limbs during reaching	Xsens MTw Awinda 3DOF IMU	60 Hz (1 participant 40 Hz)	Arms and legs (4)	**Movement organization, complexity, and coordination**: Time series of averaged acceleration	3D acceleration was reconstructed via cubic spline interpolation, reduced to 1D magnitude, smoothed with a 25 Hz low-pass Butterworth filter. And s synchronized with manual video coding using Diagonal Cross-Recurrence Quantification Analysis (DCRQA)
(Oh et.al., 2024a) [51]	To explore if wearable sensor data derived measures collected early in infancy could be correlated to developmental status as measured by the BSID-III at 12 months of age	APDM Opal Sensor Version 2 (IMU)	20 Hz	Ankles (2)	**Leg movement characteristics:** Average leg movement rate, Peak acceleration per leg movement, Fuzzy entropy of peak acceleration per leg movement	Full-day data collected. Leg movement rate, peak acceleration, and fuzzy entropy were calculated. Statistical associations between sensor variables and BSID-III scores were assessed using median regression models
(Oh et.al., 2024b) [50]	To assess kinematic leg movements of infants in rural Guatemala at risk for developmental difficulties due to poverty, malnutrition, rural location, and other environmental factors	APDM Opal Sensor Version 2 (IMU)	20 Hz	Ankles (2)	**Leg movement characteristics:** Leg movement rates (movements per hour awake) for each leg and the average Peak acceleration per movement (m/s^2^)	Leg movement rates, peak acceleration, and sleep time were calculated from sensor data. Linear mixed analyzed associations with age, swaddling, and other covariates. Wilcoxon and Shapiro-Wilk tests were used for comparisons and normality checks. Analysis was done in STATA and R
(Iverson et.al., 2024) [29]	To examine the co-development of sitting and reaching by assessing the quality of infants’ reaching movements and postural control during supported and independent sitting in infants with and without risk of Autism Spectrum Disorder	APDM Opal Sensor (IMU)	128 hz	Wrists (2)	**Movement organization, complexity, and coordination:** Mean acceleration, Mean squared jerk, Mean reach duration (from video data) Mean Center of Pressure from Pressure Mat (not wearable sensor)	Wrist sensors and a pressure-sensitive mat were synchronized via a custom trigger box; reaching segments were selected using video-coded onset and offset times. Developmental trajectories were analyzed using HLM in R

PA = physical activity, g = gravitational acceleration, ENMO = Euclidean Norm Minus One, IMU = inertial measurement unit, AUC = area under the curve, TD = typically developing, AR= at risk for developmental delay, DS=Down Syndrome, ANOVA = analysis of variance, ASD = Autism Spectrum Disorder, FFT = Fast Fourier Transform, VM = vector magnitude, MdRQA, multidimensional recurrence quantification analysis; BiFi = bilateral activity frequency, ULFi = unilateral left activity frequency, DOF = degrees of freedom, PCA = principal component analysis, SD = standard deviation, SMA = spinal muscular atrophy, CT-R = CareToy-Revised, IMP = Infant Motor Profile, AIMS = Alberta Infant Motor Scale, FDA = functional data analysis, FLM = Functional Linear Models, DCRQA = diagonal cross-recurrence quantification analysis, BSID-III = Bayley Scales of Infant Development, Third Edition, HLM = Hierarchical Linear Modeling

#### Physical Activity (PA).

Seven studies used wearable accelerometers to quantify infants’ daily PA [16,17,23,30,31,46,47]. These studies derived standardized metrics from raw acceleration including ENMO (Euclidean Norm Minus One) [46,47], signal vector magnitude (SVM) [31], vector magnitude counts [17,23], activity counts per minute [16], and accelerometer-derived prone-on-floor minutes for tummy time [30]. These sensor-based measures were then applied for several distinct purposes. Three studies used objective PA values to identify correlates of PA (i.e., demographic, behavioral, or environmental factors) [23,31,46]. One study examined the relationship between PA and motor development in infants with and without Down syndrome [16]. Another used PA to evaluate associations with adiposity across infancy [17], while a separate study assessed bi-directional associations between PA and growth indicators [47]. Finally, objective accelerometer data were used to validate parent-reported estimates of infant PA/tummy time [30].

#### Leg movement characteristics.

Across eight studies, wearable sensors were used to quantify infants’ spontaneous or task-elicited leg movements using metrics such as movement frequency, movement rate per hour awake, peak acceleration, movement duration, coordination features, barcoded multidimensional movement states, and entropy-based measures. Several studies applied acceleration-derived features to distinguish typically developing infants from those at risk for developmental delay and to examine whether early kinematic signatures predicted later developmental outcomes [12,14,26,51]. Additional work characterized developmental trajectories or assessed how environmental, caregiving, and sociodemographic factors such as swaddling or growth status influenced daily movement rate and peak acceleration [24,50]. One case study quantified total movement counts, movement rate, and kinematic profiles to illustrate how full-day wearable sensor data can complement clinician-rated assessments in a child with spinal muscular atrophy [25]. Finally, one study focused specifically on activity intensity across sleep transitions, comparing pre- and post-nap movement intensity using an area-under-the-curve metric [18].

#### Movement organization, complexity, and coordination.

Across seven studies, wearable sensors quantified the organization, complexity, and coordination of infant limb movements during spontaneous behavior and structured tasks. Researchers measured temporal coupling and averaged acceleration time series to quantify motor overflow with the purpose of determining how involuntary co-activation reflects developing neuromotor control [40]. Long-range temporal organization and kinematic descriptors such as median and peak acceleration, movement length, and bilateral movement proportion were extracted from multi-hour recordings to characterize macro-level movement organization and to identify links with naturalistic sleep–wake cycles [33]. Frequency-based Motion Complexity Index scores and entropy-based metrics were introduced to capture full-day motor complexity as potential biomarkers for early detection of atypical developmental trajectories, including infants at elevated likelihood for autism [21,39]. In structured task assessments, investigators quantified reaching quality using mean acceleration and mean squared jerk, to describe the co-development of reaching and arms-free sitting and to compare developmental pathways in infants at typical versus elevated likelihood for autism [29]. Complementary work applied multidimensional recurrence quantification and wavelet coherence analyses during task contrasts such as rattle-shaking versus free play to measure developmental changes in coordination and flexibility, with the purpose of documenting how limb-movement complexity evolves with age, how task constraints shape coordination patterns, and how social partner interactions influence rhythmic arm movements [36,37].

#### Intervention responsiveness.

Four intervention studies used wearable sensors to quantify how infants’ motor behavior changed in response to therapeutic or enriched motor experiences. The shared purpose across these studies was to determine whether sensor-based motor outcomes were sensitive to detecting short-term changes in motor engagement, dose–response effects of intervention, or early improvements in movement quality. Collectively, these studies demonstrated that wearable sensors can capture intervention-related changes in the amount, frequency, and organization of infant motor activity, supporting their potential role as objective endpoints in early motor intervention research [15,20,22,49].

### Data processing pipelines

To interpret movement from wearable sensors, studies typically employed a rigorous, multi-step data processing pipeline; 1) **Raw data collection and pre-processing**: Motion signals were recorded at sampling frequencies ranging from 1 to 150 Hz and the raw data were converted into standardized time segments (epochs), such as 1-second or 15-second windows [23,30]. Linear interpolation was used to address any missing samples and filtering techniques were used to remove noise and outliers [32,40,51]. Pre-processing often included high-pass filtering using fourth-order Butterworth filters [18,40,46] or subtraction of the gravitational component (1g) from the vector magnitude to isolate dynamic movement from static gravitational acceleration [31], 2) **Feature extraction and derived measures**: Following pre-processing, the sensor signals were summarized using derived metrics such as average acceleration or activity counts per minute [23,28,46,47]. Threshold-based algorithms were applied to acceleration and angular velocity signals to analyze specific movements [12,13,18,20–22,24–26,28,33,50,51], while more complex classification tasks relied on statistical and domain-specific features, including measures of central tendency, variability, higher-order moments, cross-correlation, and frequency-domain characteristics [27,44,45], 3) **Analysis and classification methods**: Threshold-based classification was used to categorize activity intensity based on derived metrics like jerk or acceleration [28]. For posture and activity recognition, supervised Machine Learning (ML) models were trained on the extracted feature vectors. Common algorithms include Random Forest, Support Vector Machines (SVM), k-Nearest Neighbors (k-NN), and Gaussian Mixture Models (GMM) [13,14,27,32]. More recent studies implemented Deep Learning architectures, such as Convolutional Neural Networks (CNNs) combined with recurrent layers (GRU/RNN) or residual blocks [35,44]. Some models enhanced performance using self-supervised pre-training to learn robust features from large quantities of unlabeled data before fine-tuning [35,38]. In addition, unsupervised methods were reported, such as an end-to-end deep model using an autoencoder for pre-processing and dimension reduction, followed by K-means clustering on the latent vectors [43].

## Discussion

This scoping review synthesized evidence from 41 studies examining the technical features and clinical applications of wearable sensors for monitoring infant motor activity during the first year of life. Results reveal a maturing field that has progressed from early proof-of-concept work toward broader application in clinic, home, and research contexts. The geographic distribution of included studies was predominantly from North America and Europe, likely explained by stronger technological infrastructure, sustained research funding, and multidisciplinary partnerships between clinical and engineering teams in these regions. The inclusion of both typically developing infants and those with confirmed diagnoses or at elevated risk demonstrates the broad applicability of wearable sensor technology. Importantly, more studies examined at-risk infants (53.6% %) compared to typically developing infants (36.6%), demonstrating feasibility and successful translation of sensor technology to clinical populations. This emphasis on diverse infant groups reflects a commitment to understanding developmental motor trajectories across the full spectrum of early development and aligns contemporary emphasis on health promotion and early identification, seeking to enhance habitual physical activity from the earliest developmental periods to reduce risks of obesity and delayed motor skill acquisition [52–54].

### Wearable sensor technology characteristics

Studies predominantly used triaxial accelerometers and inertial measurement units, and the hardware selection, placement, and sampling frequency decisions were increasingly driven by specific measurement objectives. Ankle and leg placement were used in studies quantifying spontaneous lower extremity movement and general activity levels, whereas wrist or arm placement was prioritized for examining upper extremity function, reaching behavior, and asymmetry. Trunk, chest, or hip placement enabled detection of whole-body movement and postural transitions. The number of sensors used per infant ranged from one to six, with greater sensor arrays providing enhanced accuracy and precision for differentiating gross motor from fine motor movements [34,36] or for complex classification of infant movement states [13,32,33,35,40,44,48]. An emerging trend was the integration of sensors into smart garments such as specialized sensor suits and jumpsuits, which may improve compliance and data quality compared to externally attached devices [13,19,32,35,38,42]. Sampling frequencies were similarly matched to assessment targets, with lower rates (1–20 Hz) employed for studies quantifying overall PA levels or prolonged postural stability, where rapid changes are less critical and battery life or data storage become practical considerations [12,14,18,19,21,22,25,26,28,45,50,51]. Mid-range frequencies (20–50 Hz) were common in studies classifying discrete movement states (e.g., sitting, standing, crawling) or quantifying limb movement frequency [17,30,41,43,44,49]. Higher frequencies (50–150 Hz) were reserved for studies requiring fine kinematic resolution, such as detecting movement asymmetries, quantifying reach quality through jerk metrics, or analyzing coordination patterns [13,15,20,23,24,27,29,31–38,40,46,48]. Data processing followed increasingly sophisticated pipelines incorporating machine learning and deep learning architectures, representing a shift from earlier parametric approaches. Features extracted also extended beyond basic acceleration and frequency metrics to include sample entropy, spectral periodicity, coordination indices, and movement complexity measures that show promise for discriminating early motor deficits and training predictive models.

### Sensor performance and validation rigor

Direct comparison across validation studies is limited by considerable methodological heterogeneity. Reference standards ranged from video annotation to clinical assessment tools, device-to-device comparison, and caregiver or environmental-motion discrimination. Studies also differed in sensor modality, category definitions, validation procedures, and reported metrics. Consequently, strong validation findings should be interpreted in light of the specific measurement framework used rather than as evidence of uniform validity across all contexts.

Collectively, the validation findings indicate that wearable-sensor systems show the strongest and most consistent evidence for quantifying infant posture, while classification of dynamic movement and clinically meaningful movement patterns remains more variable. When both outcomes were evaluated within the same study, posture was classified more accurately than movement [32,35]. Estimates of time spent in posture also showed strong correspondence with video reference standards [19,27,35]. This pattern is plausible because postures are relatively sustained, orientation-dependent states that produce more stable sensor signatures. In contrast, movement categories are transient, variable in amplitude and coordination, may occur within the same posture.

Movement performance also varied by movement type rather than uniformly by movement count. A three-category movement scheme performed no better than six- to nine-category schemes [13, 32, 35]. Within movement classification, accuracy declined further as category granularity increased: proto-form movements (early, undifferentiated motor patterns) were classified less reliably than more distinct states such as roll or transition [35], and low-intensity movements were harder to detect than higher-intensity ones [28]. Thus, an algorithm that performs well for estimating broad activity states or total time in behavioral categories may not necessarily be suitable for identifying specific movement types relevant to motor-development research. Future studies should report class-specific precision, recall, sensitivity, F1 scores, and confusion matrices, in addition to overall accuracy, to identify systematic misclassification of clinically meaningful behaviors.

Three studies assessed whether sensor-derived movement measures could distinguish infants already classified as higher-risk or lower-risk by an independent clinical reference standard (example GMA, HAI) [34,41,44]. Discrimination performance was strong across all three, with AUC values of 0.88–0.96 in two studies [34,41] and classification accuracy of 95.3–99.9% in the third [44]. This design tests whether the sensor measure agrees with a pre-established clinical classification, not whether the sensor measure could classify an infant independently of that reference standard. A related study found that the proportion of accelerometer parameters differing between infants with normal and abnormal general movements increased with postmenstrual age [48], suggesting these measures may become more informative later in the age range studied. Together, these findings support the use of sensor-derived movement measures alongside an existing clinical reference standard, rather than as a standalone screening tool. A foundational validation requirement, addressed by two studies, is determining whether sensor-derived signals reflect infant-generated movement rather than movement resulting from caregiver holding or handling [11,42]. Establishing infant-specific signal validity is an important prerequisite for subsequent movement classification and risk-discrimination analyses, as the interpretation of these downstream measures depends on accurately distinguishing infant movement from externally imposed motion.

### Clinical translation and applications

Most studies focused on applications rather than validation, with majority taking place in home rather than laboratory or clinic settings. Home-based sensor use enables repeated assessments that can better characterize intra-individual variability, diurnal patterns, and responses to naturalistic environmental affordances [55]. For clinicians, home-based data can reveal discrepancies between capacity (what an infant can do in optimal conditions) and performance (what an infant typically does in daily life). Such discrepancies may have important implications for intervention planning and family support. Application studies addressed three primary domains. First, studies quantified daily physical activity in infants and their correlates, providing insights into naturalistic activity patterns and their relationships with growth, adiposity, and motor milestone attainment [16,17,23,30,31,46,47]. These findings have direct implications for anticipatory guidance and health promotion, particularly in relation to activity exposure and positioning practices such as tummy time. Second, studies characterized spontaneous movement patterns and movement complexity, aiding researchers in identifying early motor signatures associated with later developmental outcomes, with promise for early identification of conditions like cerebral palsy or autism spectrum disorder [12,14,15,18,21,24–26,29,33,36,37,39,40,50,51]. Third, intervention studies, although fewer in number, showed that wearable sensors are sensitive to short-term changes in motor behavior following therapeutic input, supporting their potential use as objective outcome measures and tools for personalized intervention planning [20,22,49].

## Limitations

This integrative review has several limitations that should be considered when interpreting the findings. First, most studies were conducted in high-income countries with limited work addressing implementation in low-resource settings, constraining generalizability to diverse global contexts. Second, few studies established population-specific classification thresholds despite known differences in movement patterns between typically developing infants and those with motor impairments. Developing condition-specific algorithms and reference standards should be prioritized to ensure measurement validity across diverse populations. Third, while many studies demonstrated strong technical performance, fewer evaluated long-term adoption, caregiver burden, or sustainability of sensor use over extended periods. Fourth, substantial heterogeneity existed across wearable devices, sensor placements, sampling frequencies, algorithms, and outcome metrics, limiting direct comparison and precluding quantitative synthesis. Fifth, by limiting inclusion to studies published from 2019 through 2024, this review may not have captured earlier foundational Phase I–II studies that informed the development of the later Phase III–IV application of some wearable-sensor systems. In addition, because the literature search was completed in 2024 according to the prospectively registered protocol, more recent studies were not captured. Finally, the review focused on technical features and applications but did not systematically evaluate family perspectives regarding acceptability or burden associated with home-based sensor use. Despite these limitations, this review provides a comprehensive synthesis of recent advances in wearable sensing for infant motor activity, highlighting both technological progress and critical gaps that must be addressed in future research to support equitable, family-centered implementation.

## Multidisciplinary implications and future priorities

This review highlights several technical priorities for future sensor development. First, greater consensus is needed on minimum technical specifications, such as sensor type, sampling frequency, placement, and signal processing requirements, that are appropriate for different infant motor measurement objectives. Multidisciplinary stakeholder efforts could establish minimum reporting standards, core outcome sets, and recommended validation protocols to facilitate cross-study comparison and accelerate clinical translation. Second, collaborative development and dissemination of open-source processing pipelines would reduce redundant work, enable systematic algorithmic comparison, and lower barriers for clinicians and researchers without extensive computational expertise. Third, user-centered design must be prioritized. Infants are uniquely challenging for wearable technology as they cannot self-report discomfort, grow rapidly (affecting sensor fit), and frequently remove or mouth devices. Developing customized garments that integrate sensors may improve compliance and data quality compared to externally attached devices. Engaging families and clinicians through participatory design methods could yield devices better suited to real-world use. Fourth, while naturalistic data collection offers ecological validity, it introduces challenges including missing data from sensor removal or technical failures and caregiver handling artifacts that introduce significant noise. Future work should balance ecological validity with robust quality control metrics and best practices for home-based deployment.

## Conclusion

This scoping review demonstrates strong validation evidence, predominant use in naturalistic home settings, and successful application of wearable sensors to measure motor activity across diverse infant populations. For clinicians, wearables can serve as complementary measures to traditional clinical evaluation for objective physical activity assessment, early identification and monitoring of developmental concerns, and evaluation of intervention outcomes. For researchers, wearables offer the ability to obtain dense datasets on infant motor behaviors, enabling investigations of developmental dynamics, individual differences, and environmental influences at high temporal and behavioral resolution.

## Supporting information

S1 Table PRISMA-ScR checklist. (DOCX)

S2 Table PubMed search strategy. (DOCX)
