# Supplementary material for: Recent trends of infant motor activity estimation using wearable sensors: A scoping review
Source: PLOS Digit Health. 2026 Nov 6;5(11):e0001809. doi: 10.1371/journal.pdig.0001809 (PMC13743042; doi:10.1371/journal.pdig.0001809)
Supplement: Supplementary file 2 [file pdig.0001809.s002.docx]

**S2 Table**

**PubMed search strategy**

Conducted on November 13, 2024

| **Search #** | **Query** | **Results Retrieved** |
| --- | --- | --- |
| #1 | "infant" [MeSH Terms] OR "infant" [All Fields] OR "infants" [All Fields] OR "infant s" [All Fields] | 1,446,151 |
| #2 | "wearability" [All Fields] OR "wearable" [All Fields] OR "wearables" [All Fields] OR "sensor" [All Fields] OR "sensor s" [All Fields] OR "sensoric" [All Fields] OR "sensorics" [All Fields] OR "sensoring" [All Fields] OR "sensorization" [All Fields] OR "sensorized" [All Fields] OR "sensors" [All Fields] OR "accelerometer" [All Fields] OR "accelerometer s" [All Fields] OR "accelerometers" [All Fields] | 325,376 |
| #3 | "activable" [All Fields] OR "activate" [All Fields] OR "activated" [All Fields] OR "activates" [All Fields] OR "activating" [All Fields] OR "activation" [All Fields] OR "activations" [All Fields] OR "activator" [All Fields] OR "activator s" [All Fields] OR "activators" [All Fields] OR "active" [All Fields] OR "actived" [All Fields] OR "actively" [All Fields] OR "actives" [All Fields] OR "activities" [All Fields] OR "activity s" [All Fields] OR "activitys" [All Fields] OR "exercise" [MeSH Terms] OR "exercise"[All Fields] OR "activity"[All Fields] OR ("patient positioning" [MeSH Terms] OR ("patient" [All Fields] AND "positioning" [All Fields]) OR "patient positioning" [All Fields] OR "positioning" [All Fields] OR "position" [All Fields] OR "position s" [All Fields] OR "positional" [All Fields] OR "positioned" [All Fields] OR "positionings" [All Fields] OR "positions" [All Fields]) OR ("movement"[MeSH Terms] OR "movement" [All Fields] OR "movements" [All Fields] OR "movement s" [All Fields]) | 8,006,522 |
| #4 | #1 AND #2 AND #3 | 940 |
| #5 | #4 and 2019/11/13:2024/11/13[Date - Publication] | 398 |

*Date of publication restrictions applied
